# Magnetic Resonance Imaging of the Lumbar Spine: Recommendations for Acquisition and Image Evaluation from the BACPAC Spine Imaging Working Group

**DOI:** 10.1093/pm/pnac130

**Published:** 2022-09-07

**Authors:** Nico Sollmann, Aaron J Fields, Conor O’Neill, Lorenzo Nardo, Sharmila Majumdar, Cynthia T Chin, Duygu Tosun, Misung Han, An T Vu, Eugene Ozhinsky, Lubdha M Shah, Richard E Harris, Remy Lobo, William Anderst, Richard Herzog, Matthew A Psioda, Christopher J Standaert, River T Price, Jeffrey C Lotz, Thomas M Link, Roland Krug

**Affiliations:** Department of Radiology and Biomedical Imaging, University of California San Francisco, San Francisco, California, USA; Department of Diagnostic and Interventional Radiology, University Hospital Ulm, Ulm, Germany; Department of Diagnostic and Interventional Neuroradiology, School of Medicine, Klinikum rechts der Isar, Technical University of Munich, Munich, Germany; TUM-Neuroimaging Center, Klinikum rechts der Isar, Technical University of Munich, Munich, Germany; Department of Orthopaedic Surgery, University of California San Francisco, San Francisco, California, USA; Department of Orthopaedic Surgery, University of California San Francisco, San Francisco, California, USA; Department of Radiology, University of California, Davis, Sacramento, California, USA; Department of Radiology and Biomedical Imaging, University of California San Francisco, San Francisco, California, USA; Center for Digital Health Innovation, University of California San Francisco, San Francisco, California, USA; Department of Radiology and Biomedical Imaging, University of California San Francisco, San Francisco, California, USA; Department of Radiology and Biomedical Imaging, University of California San Francisco, San Francisco, California, USA; Department of Radiology and Biomedical Imaging, University of California San Francisco, San Francisco, California, USA; Department of Radiology and Biomedical Imaging, University of California San Francisco, San Francisco, California, USA; VA Advanced Imaging Research Center, San Francisco VA Health Care System, San Francisco, California, USA; Department of Radiology and Biomedical Imaging, University of California San Francisco, San Francisco, California, USA; VA Advanced Imaging Research Center, San Francisco VA Health Care System, San Francisco, California, USA; Department of Radiology, University of Utah, Salt Lake City, Utah, USA; Department of Anesthesiology, University of Michigan, Ann Arbor, Michigan, USA; Department of Radiology, University of Michigan, Ann Arbor, Michigan, USA; Department of Orthopaedic Surgery, University of Pittsburgh, Pittsburgh, Pennsylvania, USA; Department of Radiology and Imaging, Hospital for Special Surgery, New York, New York, USA; Department of Biostatistics, Gillings School of Global Public Health, University of North Carolina at Chapel Hill, Chapel Hill, North Carolina, USA; Department of Physical Medicine and Rehabilitation, University of Pittsburgh School of Medicine, Pittsburgh, Pennsylvania, USA; Department of Biostatistics, Gillings School of Global Public Health, University of North Carolina at Chapel Hill, Chapel Hill, North Carolina, USA; Department of Orthopaedic Surgery, University of California San Francisco, San Francisco, California, USA; Department of Radiology and Biomedical Imaging, University of California San Francisco, San Francisco, California, USA; Department of Radiology and Biomedical Imaging, University of California San Francisco, San Francisco, California, USA

**Keywords:** Magnetic Resonance Imaging, Intervertebral Disc Degeneration, Low Back Pain, Paraspinal Musculature, Vertebral Endplate

## Abstract

Management of patients suffering from low back pain (LBP) is challenging and requires development of diagnostic techniques to identify specific patient subgroups and phenotypes in order to customize treatment and predict clinical outcome. The Back Pain Consortium (BACPAC) Research Program Spine Imaging Working Group has developed standard operating procedures (SOPs) for spinal imaging protocols to be used in all BACPAC studies. These SOPs include procedures to conduct spinal imaging assessments with guidelines for standardizing the collection, reading/grading (using structured reporting with semi-quantitative evaluation using ordinal rating scales), and storage of images. This article presents the approach to image acquisition and evaluation recommended by the BACPAC Spine Imaging Working Group. While the approach is specific to BACPAC studies, it is general enough to be applied at other centers performing magnetic resonance imaging (MRI) acquisitions in patients with LBP. The herein presented SOPs are meant to improve understanding of pain mechanisms and facilitate patient phenotyping by codifying MRI-based methods that provide standardized, non-invasive assessments of spinal pathologies. Finally, these recommended procedures may facilitate the integration of better harmonized MRI data of the lumbar spine across studies and sites within and outside of BACPAC studies.

## Introduction

Magnetic resonance imaging (MRI) is the imaging modality often used to depict spinal structural abnormalities that may cause low back pain (LBP). With its high soft tissue contrast, MRI allows assessment of multiple structures including intervertebral discs, nerve roots, contents of the central spinal canal, ligaments and facet joints [1–5]. While MRI is superb at identifying tissue-specific pathology along the spine, the clinical relevance of these findings is often uncertain. Thus, diagnostic MRI for LBP is only recommended in certain patients. The American College of Physicians’ guidelines for diagnostic imaging suggest immediate imaging when there are major risks (such as suspicion of cancer, spinal infection, cauda equina syndrome, or presence of severe neurological deficits), but advise deferral of imaging pending a trial of treatment in cases where there are weaker risk factors [6, 7]. Notably, only in a minority of patients can LBP be attributed to a specific pathology (e.g., vertebral fracture or disc herniation with nerve compression), while the great majority of patients are assigned a diagnosis of non-specific LBP (i.e., pain for which the distinct patho-anatomical basis cannot be determined) [7, 8].

Some lumbar MRI findings that are common in patients with LBP are also common in asymptomatic subjects, which makes it difficult to define a causal relationship in individual patients and has led to questions about the diagnostic value of such findings [9–11]. However, some lumbar MRI findings are more prevalent in patients with LBP than in asymptomatic subjects [10–12]. Those include bone marrow (BM) lesions located at the vertebral endplates, disc bulges and herniation, and spondylolysis [10–12]. Such lumbar MRI findings could potentially function as biomarkers that can distinguish patients whose pain is due to a primary nociceptive process in the spine from patients with central modulating mechanisms that can be influenced by a number of psychosocial factors as well as central sensitization (i.e., increased responsiveness of nociceptors to either normal or sub-threshold afferent input, resulting in amplified pain) [13–16].

A major focus of the Back Pain Consortium (BACPAC) Research Program is to advance knowledge of the etiology and treatment of chronic LBP by developing a better understanding of the mechanisms contributing to chronic LBP and by identifying specific therapies that are most effective in identifiable subgroups of patients. The BACPAC Spine Imaging Working Group has developed standard operating procedures (SOPs) for lumbar spine imaging exams to be used in all BACPAC studies. These SOPs include procedures to conduct imaging assessments with guidelines for standardizing the collection, reading/grading, and storage of images across studies and sites within and outside of BACPAC. Our aim was to harmonize imaging protocols and implement a comprehensive evaluation scheme for identifying potential imaging biomarkers of LBP.

## Imaging Protocol

### Setup and Sequence Planning

Patients eligible for BACPAC studies will undergo an MRI examination (acquisition duration <60 min, depending on the distinct pulse sequence protocol) that is performed in a supine position using a spine coil or a table-embedded coil array. Following an initial phase of sequence optimization at each BACPAC site (including testing and determination of flip angles, fat suppression [FS] parameters, field of view [FOV] sizes, and subject/coil configurations for an optimized signal-to-noise ratio), MRI acquisition with a standardized pulse sequence protocol is performed. The recommendations of the BACPAC Spine Imaging Working Group regarding the distinct pulse sequence protocols refer to non-contrast MRI using a 3-Tesla or 1.5-Tesla MRI scanner. Furthermore, recommended is the use of the available clinical protocol for lumbar spine imaging as a starting point and make adjustments, if necessary, as described below.

Sequences will be manually planned (i.e., positioning and angulation of the FOV) using an initially acquired survey scan. At least the entire lumbosacral spine in cranio-caudal direction as well as the complete diameter of the vertebrae, facet joints (FJs), neuroforamina, and paraspinal musculature in transverse direction (and for predefined sequences also the sacroiliac joints [SIJs]) will be covered. When magnetic resonance neurography (MRN) of the lumbosacral plexus is additionally acquired, the anterior body coil (or torso coil) array should be used in addition to the spine coil or table-embedded coil array, and the FOV should cover the area from L1/L2 through the ischial tuberosities.

### Imaging Sequences

Regarding imaging with T1- and T2-weighted sequences, the BACPAC Spine Imaging Working Group recommends a minimal pulse sequence protocol that will be acquired in each patient, supplemented by additional sequences based on technical infrastructure at the acquisition site:

- Minimal recommended sequence protocol:
  - Sagittal T2-weighted fast spin-echo (FSE) sequence with FS: sagittal T2 FS
  - Sagittal T1-weighted FSE sequence without FS: sagittal T1
  - Axial T2-weighted FSE sequence without FS: axial T2
- Additional recommended sequences (if feasible):
  - Sagittal T2-weighted FSE sequence without FS: sagittal T2
  - Sagittal T1-weighted FSE sequence without FS (covering the SIJ): sagittal T1
  - Coronal T1-weighted FSE sequence without FS (covering the SIJ): coronal T1
  - Axial T1-weighted FSE sequence without FS: axial T1
  - MRN lumbosacral plexus: three-dimensional (3D) T2-weighted FSE sequence with FS: axial MRN

The specific parameters for these T1- and T2-weighted FSE sequences used for the MRI machine (GE 3 T Discovery MR750; GE Healthcare, Waukesha, WI, USA) and coil (8-channel phased-array spine coil; GE Healthcare, Waukesha, WI, USA) at one site of image acquisitions are shown in Table 1. Due to variations of pulse sequence implementations and hardware specifics between different MRI machines or vendors, ranges for pulse sequence protocols are proposed and shown in Table 2.

**Table 1.** Pulse sequence parameters for conventional T1- and T2-weighted sequences (standardized protocol)

Sequence	SAG T2 FS (Lumbar)	SAG T2 (Lumbar)	SAG T1 (Lumbar)	AX T2 (Lumbar)	AX T1 (Lumbar)	SAG T1 (with SIJ)	COR T1 (with SIJ)	AX MRN (Lumbosacral)
Patient Position	Head first, supine	Head first, supine	Head first, supine	Head first, supine	Head first, supine	Head first, supine	Head first, supine	Feet first, supine
Plane	SAG	SAG	SAG	AX	AX	SAG	COR	AX
Flip Angle [°]	120	120	110	120	110	115	115	Variable Refocusing Flip Angle
TE [ms]	60	60	14.1	60	14.1	14.1	14.1	100
TR [ms]	4,551	5,035	869	8,414	594	720	595	2,500
Echo Train Length	16	18	6	16	6	6	6	100
Receiver Bandwidth [kHz/0.5FOV]	50	50	50	50	50	50	50	62.5
FOV [cm]	26	26	26	18	18	30	30	36x29
Slice Thickness [mm]	3.0	3.0	3.0	4.0	4.0	8.0	4.0	1.2
Slice Spacing [mm]	3.0	3.0	3.0	5.0	5.0	10	5.0	N/A
Frequency Matrix	384	384	384	288	288	320	384	300
Phase Matrix	224	224	224	192	192	160	224	240
Frequency Direction	A/P	A/P	A/P	R/L	R/L	A/P	R/L	R/L
NEX averages	3.0	3.0	2.0	2.0	2.0	2.0	2.0	1.0
Fat suppression	On	Off	Off	Off	Off	Off	Off	On
Acceleration	Off	Off	Off	Off	Off	Off	Off	On
Number of Slices	24	24	24	42	42	36	20	120
Scan Time [min: sec]	3:16	3:16	3:34	3:31	3:47	2:06	1:42	8:30

This table shows the sequence parameters for the conventional T1-/T2-weighted sequences for the main site’s magnetic resonance imaging (MRI) system (GE 3 T Discovery MR750; GE Healthcare, Waukesha, WI, USA). For MRN, acceleration is achieved with 2 × 1.5 × 1.4 (ky phase-encode direction × kz phase-encode direction x compressed sensing factor).

AX = axial; SAG = sagittal; COR = coronal; FS = fat suppression; TE = echo time; TR = repetition time; FOV = field of view; R/L = right/left; A/P = anterior/posterior; NEX = number of excitations (averages); SIJ = sacroiliac joint.

**Table 2.** Pulse sequence parameters for conventional T1- and T2-weighted sequences (recommended ranges)

Sequence	SAG T2 FS (Lumbar)	SAG T2 (Lumbar)	SAG T1 (Lumbar)	AX T2 (Lumbar)	AX T1 (Lumbar)	SAG T1 (with SIJ)	COR T1 (with SIJ)	AX MRN (Lumbosacral)
Patient Position	Head first, supine	Head first, supine	Head first, supine	Head first, supine	Head first, supine	Head first, supine	Head first, supine	Feet first, supine
Plane	SAG	SAG	SAG	AX	AX	SAG	COR	AX
Flip Angle [°]	105–180	105–180	105–180	105–180	105–180	105–180	105–180	Variable Flip Angle
TE [ms]	40–80	40–80	8–20	40–80	8–20	8–20	8–20	80–120
TR [ms]	≥ 2,000	≥ 2,000	500–900	≥ 2,000	500–900	500–900	500–900	1,500–2,500
Echo Train Length	15–24	15–24	3–8	15–24	3–8	3–8	3–8	60–120
Receiver Bandwidth [kHz/0.5FOV]	31–62	31–62	31–62	31–62	31–62	31–62	31–62	31–62.5
FOV [cm]	25–28	25–28	25–28	14–22	14–22	30–32	26–32	36–38
Slice Thickness [mm]	≤ 4.0	≤ 4.0	≤ 4.0	≤ 4.0	≤ 4.0	≤ 8.0	≤ 4.0	≤ 1.5
Slice Spacing [mm]	≤ 4	≤ 4	≤ 4	≤ 5	≤ 5	≤ 10	≤ 5	N/A
Frequency Matrix	320–512	320–512	320–512	256–512	256–512	320–512	256–512	256–380
Phase Matrix	192–320	192–320	192–320	192–320	192–320	160–320	192–320	200–304
Frequency Direction	A/P	A/P	A/P	R/L	R/L	A/P	R/L	R/L
NEX averages	1–4	1–4	1–4	1–4	1–4	1–4	1–4	1
Fat suppression	On	Off	Off	Off	Off	Off	Off	On
Number of Slices	≥ 12	≥ 12	≥ 12	≥ 30	≥ 30	≥ 30	≥ 12	N/A
Scan Time [min]	≤ 5 min	≤ 5 min	≤ 5 min	≤ 5 min	≤ 5 min	≤ 4 min	≤ 3 min	≤ 10 min

Due to variations in magnetic resonance imaging (MRI) machines and vendors between imaging sites, ranges for pulse sequence protocols are proposed for conventional T1-/T2-weighted sequences.

AX = axial; SAG = sagittal; COR = coronal; FS = fat suppression; TE = echo time; TR = repetition time; FOV = field of view; R/L = right/left; A/P = anterior/posterior; NEX = number of excitations (averages); SIJ = sacroiliac joint.

Of note, the preference for specific echo times (TEs) of T2-weighted FSE sequences can also vary depending on whether structures within vertebral bodies and intervertebral discs are more important (typical TE around 60 ms), or depictions of spinal cord and nerves are more important (longer TEs, 80 ms or higher). As an additional recommended sequence, dedicated 3D MRN can be acquired in selected cases to delineate the peripheral nerves of the lumbosacral plexus for diagnosis and characterization of neuropathy (e.g., due to compressive effects in relation to a herniated disc) [17–22].

## Evaluation of Imaging Data

Based on the MRI data acquired, we propose qualitative/semi-quantitative image evaluation (e.g., using ordinal rating scales) using structured reporting with a predefined scoring system that incorporates the different structures and related grading of pathology as captured by the FOV of the T1- and T2-weighted sequences (Modic-type endplate changes, endplate defects, intervertebral disc changes, FJ and SIJ changes, and stenosis).

### Modic Changes

Modic-type endplate changes summarize three categories based on specific signal characteristics of vertebral BM adjacent to the endplates in MRI [23]. Most commonly, type 1 changes are considered an inflammatory, fibrovascular stage and may indicate an ongoing active degenerative process (e.g., disruption and fissuring of endplates and formation of granulation tissue) [23, 24]. Type 2 changes are related to fatty degenerative remodeling processes, with replacement of red by yellow BM within the vertebrae [23, 24]. Furthermore, type 3 changes reflect the sclerotic stage [23, 24]. Related to their suggested pathophysiological entity, the three different types of Modic-type endplate changes differ in their appearances using the combination of sagittal T1- and T2-weighted sequences, with Modic changes type 1 typically showing hypointense signal on T1- and hyperintense signal on T2-weighted images, Modic changes type 2 showing hyperintense signal on T1- and hyper- or iso-intense signal on T2-weighted images without FS (typically relatively hypointense on T2-weighted images with FS), and Modic changes type 3 showing hypointense signal on T1- and T2-weighted images [23, 25, 26]. However, mixed types can be observed frequently for one lumbar segment (e.g., presence of signal characteristics indicative of Modic changes type 1 and type 2), and categorization may be done by reporting the dominant type.

**Figure 1.**
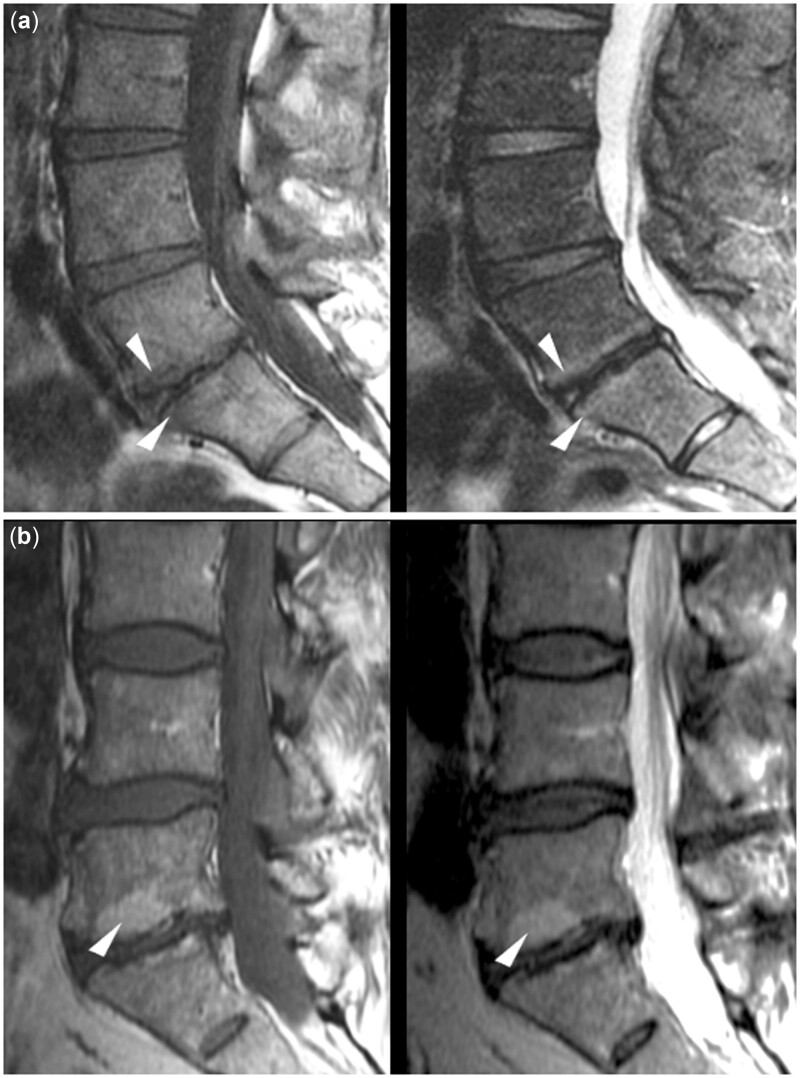
Modic-type endplate changes. Examples of Modic-type endplate changes, which can be identified according to characteristic signal alterations in T1- and T2-weighted images. Modic changes type 1 are typically hypointense on T1- and hyperintense on T2-weighted images (a; arrowheads), and Modic changes type 2 are hyperintense on T1- and hyper- or iso-intense on T2-weighted images without fat suppression (b; arrowheads). Modic type 3 changes are primarily characterized by sclerotic changes (not shown).

Regarding reliability of assessments of Modic-type endplate changes on lumbar MRI, literature reports on varying but substantial to excellent intra-rater agreement (κ  >  0.70) and inter-rater agreement (κ  >  0.78) [27, 28]. Furthermore, the diagnostic performance of the Modic classification for discography-concordant pain yielded a specificity of at least 95% [29–33]. However, corresponding sensitivity was reported to be rather low and variable, with values ranging between 14% and 48% [29–33]. In addition to classifications into the three types of Modic-type endplate changes, the extent of changes in relation to vertebral body height and diameter is determined (Table 3). Example cases for Modic-type endplate changes are shown in Figure 1.

**Table 3.** Modic-type endplate changes

	0	1	2	3	4	Sequences
Modic Changes	absent	present				
Modic Type		T1: hypointense T2: hyperintense T2 FS: hyperintense	T1: hyperintense T2: hyperintense T2 FS: iso-/hypo-intense	T1: hypointense T2: hypointense T2 FS: hypointense		SAG T2 SAG T1 SAG T2 FS
Vertebral Body Height		Localized at endplate only	Less than 25% of vertebral body height	25 to 50% of vertebral body height	More than 50% of vertebral body height	SAG T2 SAG T1 SAG T2 FS
Affected Endplate Area (largest diameter in a single section)		Less than 25% of endplate area	25 to 50% of endplate area	More than 50% of endplate area		SAG T2 SAG T1 SAG T2 FS

This table provides the scoring scheme for Modic changes and the sequences used for evaluation of different categories. In case that Modic changes are detected, the type (Modic type I—III) as well as the spatial characteristics of changes with respect to vertebral body height and vertebral endplate area should be graded.

### Endplate Defects

**Figure 2.**
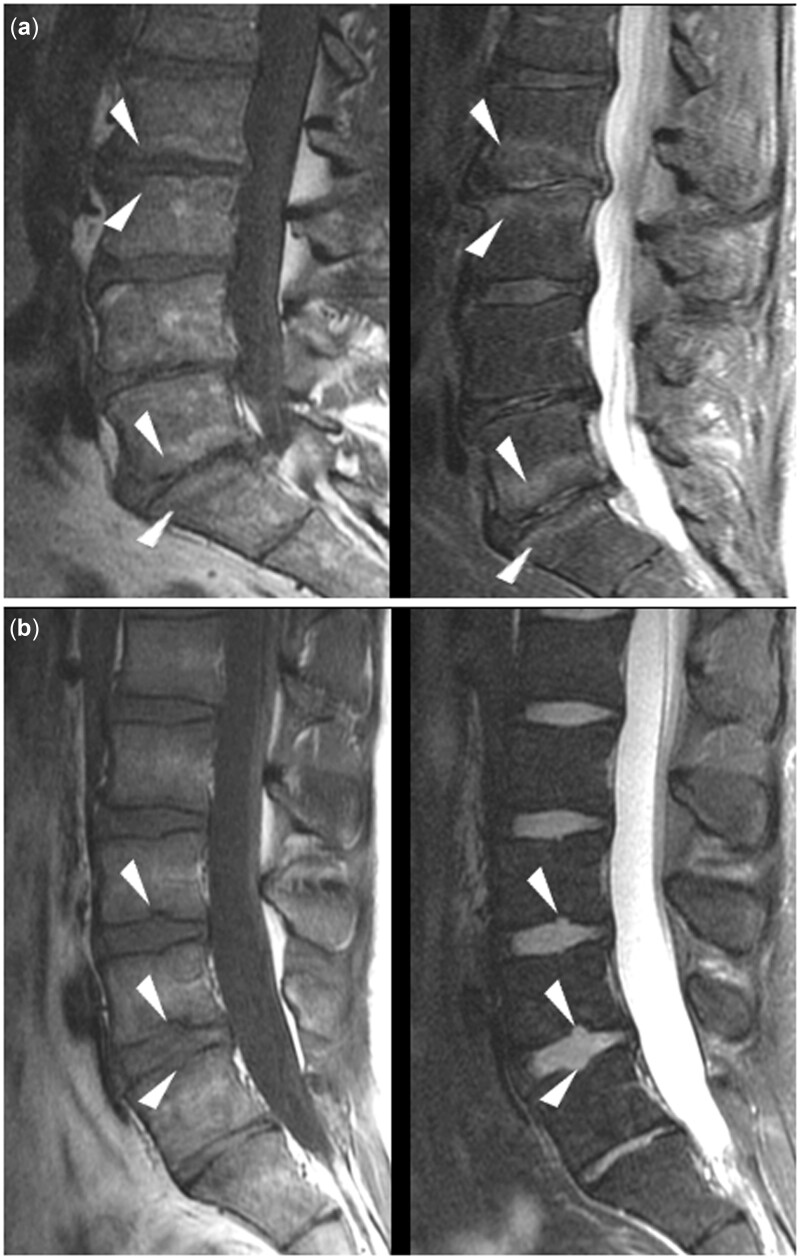
Endplate defects. Examples of endplate defects with degenerative appearance (erosive osteochondrosis) on T1- and T2-weighted sequences (a; arrowheads) and focal Schmorl’s nodes on T1- and T2-weighted sequences (b; arrowheads).

Structural changes at the vertebral endplates are scored according to their appearance into degenerative changes (e.g., erosive intervertebral osteochondrosis), Scheuermann variant, and osteoporotic fractures. Erosive intervertebral osteochondrosis entails widespread erosions of the vertebral body endplates with disc degeneration, commonly leading to findings of vacuum phenomenon within the intervertebral disc, hypointensity within the intervertebral disc on T2-weighted images, band-like subchondral edema, and fat accumulation or sclerosis for affected vertebral bodies [34]. Scheuermann variant is a developmental disorder with a hereditary predisposition component, with characteristic features of thoracic spine kyphosis >40° or thoraco-lumbar spine kyphosis >30° and vertebral wedge configuration of at least 5° at each level, involving at least three adjacent vertebral bodies according to the Sorensen criteria [35, 36]. A differential diagnosis is represented by changes of vertebral body configuration due to osteoporotic fractures, which can also appear as wedging of one or more vertebral bodies [37, 38]. However, the entity is different, with osteoporosis being characterized by low bone mineral density in combination with micro-architectural deterioration of bone tissue, thus increasing the risk for fragility fractures [39–44]. To provide further insights into the distinct appearance of endplate defects, they are further categorized according to shape as well as size and depth for a representative section (Table 4). Example cases for endplate defects are illustrated by Figure 2.

**Table 4.** Endplate defects

	0	1	2	3	4	Sequences
Endplate Defect	Absent	Present				
Type		Degenerative (e.g., erosive intervertebral osteochondrosis)	Scheuermann variant (Schmorl’s nodes); wedge-shaped deformity and increased sagittal diameter	Osteoporotic (normal sagittal diameter)	Other	SAG T1 SAG T2
Shape		Irregular, diffuse	Schmorl’s node, subchondral cyst	Sharp, angular, focal	Other	SAG T1 SAG T2
Size (largest diameter in a single section)		Less than 1/3 of endplate area	Between 1/3 and 2/3 of endplate area	More than 2/3 of endplate area		SAG T1 SAG T2
Depth (largest diameter in a single section)		Less than 25% of vertebral body height	25 to 50% of vertebral body height	More than 50% of vertebral body height		SAG T1 SAG T2

This table outlines the scoring scheme for vertebral endplate defects and the sequences used for evaluation of single categories. In case that vertebral endplate defects are detected, the type as well as the spatial characteristics of changes with respect to vertebral body height and vertebral endplate area should be graded, as well as their shapes.

### Intervertebral Disc Changes

Lumbar disc degeneration is graded according to the widely used Pfirrmann classification, which defines five grades in total according to the intervertebral disc height as well as signal characteristics shown on T2-weighted sequences [45]. Specifically, it spans from grade I for a homogeneous disc with normal height and bright hyperintense white signal intensity to grade V for an inhomogeneous disc with collapsed disc space and a hypointense black signal intensity [45]. The Pfirrmann classification for degenerative disc disease has been reported to show substantial to excellent inter-rater (κ  >  0.65) and intra-rater (κ  >  0.73) reliability [45–48]. This grading is supplemented by information on potential contact between disc material and nerve roots, or deviation or compression of nerve roots due to disc degeneration (Table 5) [49].

**Table 5.** Intervertebral disc changes

	0	1	2	3	4	5	Sequences
Degenerative Disc Disease		Homogeneous, hyperintense, normal height	Inhomogeneous, hyperintense, normal height, clear distinction annulus vs. nucleus	Inhomogeneous, gray, normal height, no clear distinction between annulus vs. nucleus	Inhomogeneous, gray to black, normal height to moderate loss, no distinction between annulus vs. nucleus	Inhomogeneous, black, > 50% height loss	SAG T2
Disc Height	Less than 10% loss (mild)	10%–50% loss (moderate)	>50% loss (severe)				SAG T2
Disc Herniation	Normal	Bulge	Protrusion	Extrusion			SAG T2 FS AX T2
Protrusion/Extrusion Location	Central	Subarticular zone	Foraminal	Extraforaminal			SAG T2
Protrusion/Extrusion Side	Left	Right					SAG T2
Annular Fissure	Absent	Present					SAG T2
Annular Fissure location	Central/ posterior	Left/ subarticular	Right/subarticular				SAG T2
Nerve Root Involvement	No nerve root contact	Nerve root—left contact without deviation	Nerve root—left deviation and compression	Nerve root—right contact without deviation	Nerve root—right deviation and compression	Bilateral	SAG T1 SAG T2 AX T2

This table shows the scoring scheme for intervertebral disc changes and the sequences used for evaluation of respective categories. In case that degenerative disc disease is detected, its characteristics including signal, height, herniation specifics, and nerve root involvement should be graded, together with screening for an annular fissure.

**Figure 3.**
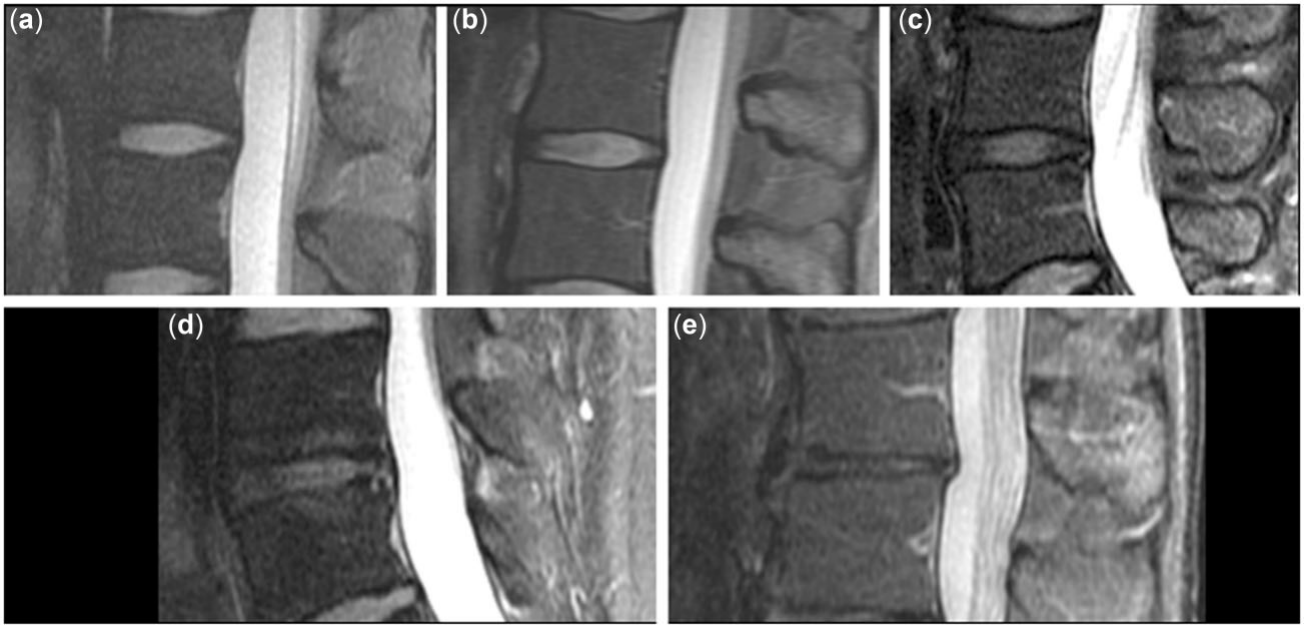
Degenerative disc disease. Examples for grading of intervertebral discs: homogeneous, hyperintense, normal height of disc (a; Pfirrmann grade I); inhomogeneous, hyperintense, normal height, clear distinction annulus versus nucleus (b; Pfirrmann grade II); inhomogeneous, gray, normal height, no clear distinction between annulus versus nucleus (c; Pfirrmann grade III); inhomogeneous, gray to black, normal height to moderate loss, no distinction between annulus versus nucleus (d; Pfirrmann grade IV); and inhomogeneous, black, > 50% height loss (e; Pfirrmann grade V).

**Figure 4.**
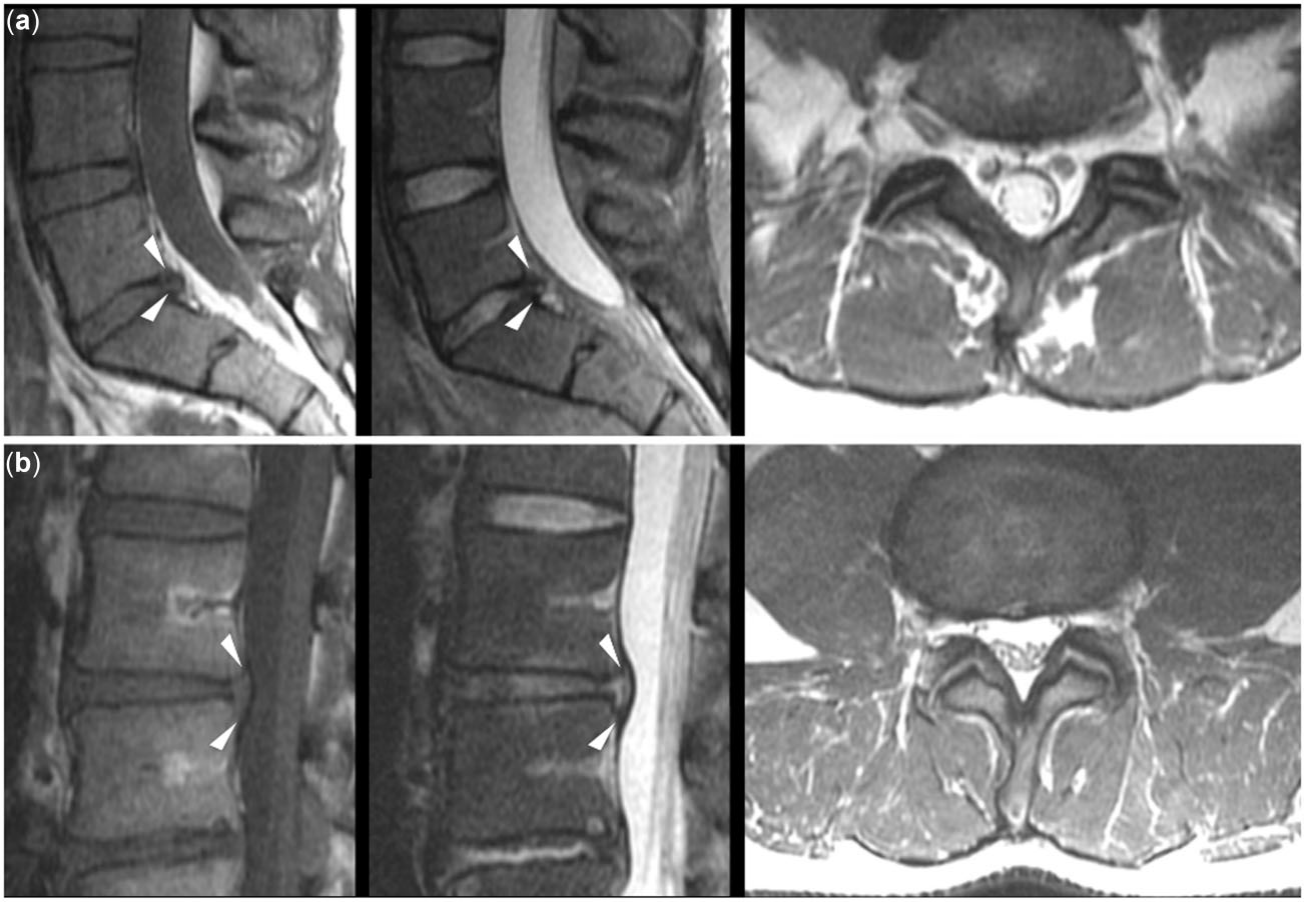
Disc herniation. Example cases for disc protrusion (a; arrowheads) and disc extrusion (b; arrowheads) on sagittal T1- and T2-weighted sequences and axial T2-weighted sequences. A disc protrusion is considered in case of a focal herniation of disc material beyond the margins of the adjacent vertebral bodies, while a disc extrusion is a focal herniation of disc nuclear material through an annular defect. In both presented cases, absent to minor resulting constriction of the thecal sac is shown on axial sequences.

Furthermore, location and morphology of disc herniation is classified according to shape characteristics, including bulge, protrusion, and extrusion [50, 51]. Specifically, bulge is defined as annular tissue that projects beyond the margins of the adjacent vertebral bodies, affecting more than 90° of circumference [50, 51]. A disc protrusion is a focal herniation of disc material beyond the margins of the adjacent vertebral bodies (over less than 90° of circumference and with a base that is wider than the dome) [50, 51]. In addition, a disc extrusion is a focal herniation of disc nuclear material through an annular defect (remaining in continuity with the disc and with a base narrower than the dome) [50, 51]. Additionally, the absence or presence of an annular fissure and its location is taken into consideration, which is commonly characterized by a zone of high signal on T2-weighted images at the lateral edges of the annulus (Table 5) [50, 51]. Example cases for degenerative disc disease are provided by Figures 3 and 4.

### Facet and Sacroiliac Joint Changes

**Figure 5.**
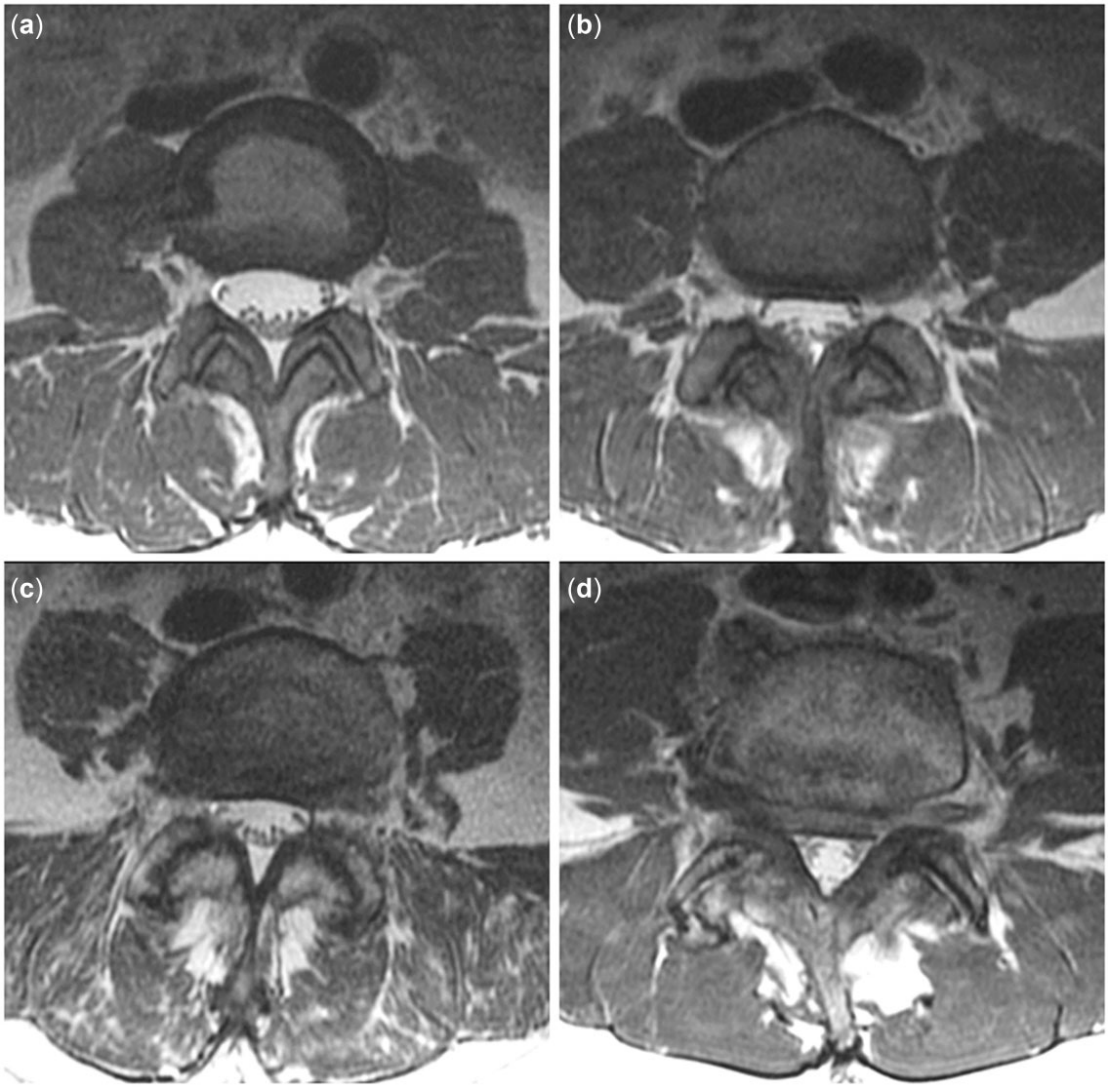
Facet joint (FJ) degenerative changes. Examples for grading of FJ changes: normal appearance (a); narrowing, small osteophytes or hypertrophy (b); narrowing, moderate osteophytes or hypertrophy (c); and narrowing, large osteophytes or severe hypertrophy (d).

Arthropathies of the FJ or SIJ are typically characterized by joint space reductions, hypertrophy of articular processes, and formation of osteophytes. For the FJs, grading into four groups according to these characteristics is made with respect to the grading approaches by Fujiwara and Pathria and their coworkers (Table 6) [52, 53]. For grading of FJ arthropathy using MRI, a rather weak inter-rater reliability (κ > 0.40) has been reported, although this grading has been widely used [46, 54]. Example cases for FJ arthropathy grading are shown in Figure 5.

**Table 6.** Facet and sacroiliac joint changes

	0	1	2	3	Sequences
Facet joint (FJ)					
Joint Space	None	Narrowing, small osteophytes or hypertrophy	Narrowing, moderate osteophytes or hypertrophy	Narrowing, large osteophytes or severe hypertrophy	SAG T2 FS AX T2 AX T1
Joint Fluid	Absent	Present			AX T2 FS
Sacroiliac joint (SIJ)					
Appearance	Normal	Abnormal			SAG T2 FS COR T1
Joint Space	Normal	Degenerative	Erosions		COR T1
Bone Marrow	Normal	Edema	Fatty transformation		SAG T1 COR T1
Bone	Normal	Insufficiency fractures	Osteitis condensans ilii	Other	SAG T1 COR T1
Lumbosacral Transitional Vertebrae Type	None	Large transverse processes (>2.1 cm)	Large transverse process articulating with the sacrum	Large transverse process fused with sacrum	SAG T1 SAG T2 FS COR T1
Lumbosacral Transitional Vertebrae Location	Bilateral	Left	Right	Asymmetric	SAG T1 SAG T2 FS COR T1

This table illustrates the scoring scheme for changes of the facet joints (FJs) and sacroiliac joints (SIJs) and the sequences used for evaluation of different categories. In addition, presence and degree of lumbosacral transitional vertebrae (LSTV) should be assessed.

For the SIJ, edematous or fatty changes are evaluated, together with presence or absence of insufficiency fractures or alterations suggestive for osteitis condensans ilii [55–57]. Furthermore, the presence and degrees of lumbosacral transitional vertebrae (LSTV) are evaluated based on a rating scheme modified from that of Castellvi et al. (Table 6) [58, 59]. The inter-rater reliability was demonstrated to be excellent for the detection (κ  =  0.93) and classification (κ  =  0.83) of LSTV based on MRI [60].

### Stenosis

**Figure 6.**
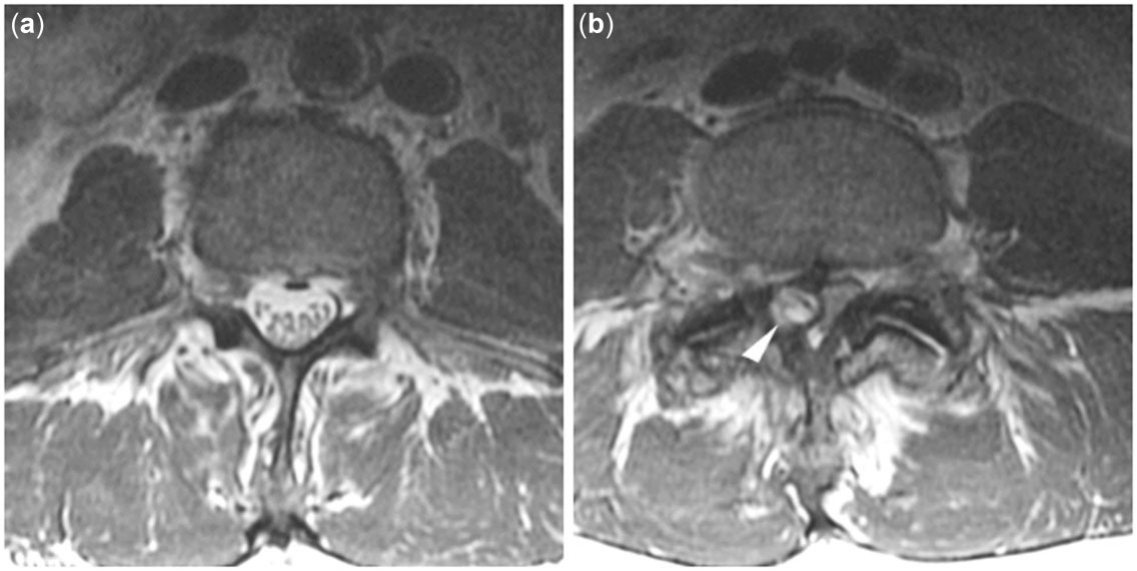
Spinal canal stenosis. Normal configuration and diameter of the spinal canal (a) and severely narrowed spinal canal with complete loss of cerebrospinal fluid (CSF) signal due to degenerative changes with a synovial cyst (b; arrowhead).

Evaluation of central canal stenosis is made according to the contour of the thecal sac and amount of cerebrospinal fluid (CSF) space around the spinal cord. In detail, discrimination is made between no thecal sac constriction, mild constriction (minimal loss of CSF around rootlets), moderate grade (CSF diminished but present), and severe grade of central canal stenosis with complete loss of CSF signal (Table 7). In addition, features indicative of congenital narrowing of the spinal canal (i.e., shorter pedicular length and related smaller cross-sectional spinal canal area) are documented (Table 7) [61]. Example cases for spinal canal narrowing are given by Figure 6.

**Table 7.** Stenosis

	0	1	2	3	Sequences
Central Canal	No thecal sac constriction	Mild constriction, minimal loss of CSF around rootlets	CSF diminished but present	Complete loss of CSF	SAG T2 FS AX T2
Congenital Narrowing of Central Canal	No	Yes			SAG T2 FS AX T2
Subarticular Zone Stenosis Characteristics	No nerve root contact	Nerve root contact without deviation	Nerve root compression		SAG T2 FS AX T2
Subarticular Zone Stenosis Side	Left	Right	Bilateral		SAG T2 FS AX T2
Neuroforaminal Stenosis Characteristics	Normal epidural fat	Mild—slight deformity of epidural fat, still completely surrounding the nerve root	Moderate—marked deformity of epidural fat, only partially surrounding nerve root	Severe—obliteration of epidural fat	SAG T1 SAG T2 FS
Neuroforaminal Stenosis Side	Left	Right	Bilateral		SAG T1 SAG T2 FS

This table outlines the scoring scheme for central canal stenosis and neuroforaminal stenosis or stenosis at the subarticular zone and the sequences used for evaluation of such stenotic changes.

With regard to the course of the exiting spinal nerve roots, contact to surrounding compressive structures (e.g., herniated intervertebral discs or FJ arthropathy) with or without deviation and side are evaluated for the subarticular zone. For the neuroforamina, stenosis is classified as mild (i.e., slight deformity of epidural fat, still completely surrounding nerve root), moderate (i.e., marked deformity of epidural fat, only partially surrounding nerve root), and severe (i.e., obliteration of epidural fat), according to changes in epidural fat configuration as a surrogate of compression at the level of the neuroforamina (Table 7) [62]. Inter-rater agreement for the grading of neuroforaminal stenosis has been reported to be excellent (κ  >  0.90), with slightly lower agreement for intra-rater comparisons (κ  >  0.79) [62].

## Discussion

Within the BACPAC Research Program, MRI studies are being used to facilitate better understanding of pain mechanisms in chronic LBP by developing, establishing, and translating standardized MRI-based methods. A main goal is to identify specific phenotypes of LBP that would enable reliable guidance for patient selection for certain treatment regimens and to predict responses to treatments, with the ultimate goal to improve clinical management and therapy.

The SOPs we have described address two sources of variability in MRI for patients with LBP—image acquisition and image interpretation. While lumbar MRI can be a powerful modality for LBP assessment in cases where imaging is warranted, imaging protocols and MRI systems vary greatly between sites. Imaging within the BACPAC Research Program implements a standardized procedure for scanning, whilst technical variations and particularities between scanners at different sites are acknowledged by recommending ranges for pulse sequence protocols. The herein presented SOPs may help to better integrate data across studies and sites to facilitate robust analyses and inferences using much larger datasets than would otherwise be available. Furthermore, evaluation of better harmonized MRI data of the lumbar spine across studies and sites within and outside of BACPAC studies is supported by these SOPs, given that variability in used equipment and pulse sequence protocols could be reduced. For evaluation of conventional MRI using T1- and T2-weighted sequences in LBP, structured reporting is established to reduce inconsistent reporting or subjective misinterpretations of findings during image reading. Structured reporting uses predefined terms and formats to generate a radiological report, thus ensuring a high degree of standardized and organized information that is presented in template context [63, 64]. Structured reporting is not broadly utilized in LBP, but it may help to reduce variations during image reading, and it could thus provide condensed information relevant for structural alterations potentially linked to pain perception and for identification of imaging-based biomarkers.

Potential biomarkers of LBP that are commonly detected on routine clinical scans include BM lesions at the vertebral endplates, disc bulges and herniation, and spondylolysis [10–12]. The proposed comprehensive scoring system is based on visual reading of T1- and T2-weighted sequences with structured reporting. A strength is that this scoring system integrates previously established grading schemes within a standardized approach. The majority of such schemes has demonstrated at least substantial inter- and/or intra-rater reliability (e.g., classification of Modic-type endplate changes or Pfirrmann grading for degenerative disc disease) [27, 28, 45–48]. On the other hand, potential weaknesses include the qualitative/semi-quantitative nature of analyses by visual image reading and bias towards only structural findings rather than compositional findings. Related to this, the relationships between pathologies evaluated by these established grading schemes and pain might be variable, and thus, the clinical implications are unclear. For instance, it has been reported that Modic-type endplate changes have inconsistent associations with pain [12, 65–70]. Inconsistencies may stem from several sources, including imprecise description of Modic-type endplate changes for clinical reports, utilization of MRI equipment that introduces grading bias, or acquisition of imaging sequences that inadvertently misrepresent these changes [71]. Specifically, omission of basic methodological details and, most notably, restriction to mainly qualitative/semi-quantitative, non-standardized evaluation of MRI data for Modic-type endplate changes make it difficult to draw more final conclusions about the correlation of LBP and vertebral endplate pathology [71].

Several novel MRI-based methods are undergoing research and development that may allow detection of biomarkers not seen on conventional lumbar MRI on the way to improved understanding of LBP mechanisms. For example, magnetic resonance spectroscopy (MRS) was used to quantify spectral features related to intervertebral disc structure and acidity, and results from MRS correlated well with provocative discography, thus could potentially support improved surgical outcomes among patients with chronic LBP [72]. Furthermore, ultra-short echo-time (UTE) imaging and chemical shift encoding-based water-fat MRI (CSE-MRI) have been proposed to be more sensitive to endplate damage or BM pathology and allow both to be quantified, yet without the need for specialized MRI hardware or application of contrast agents [73–79]. Structural damage of the endplate is believed to trigger endplate neo-innervation and painful endplate BM lesions, and cartilage endplate fibrosis blocks nutrient transport to the disc cells, thereby hindering cell survival, disc matrix homeostasis, and regenerative potential [80, 81].

Another novel MRI-based approach that may provide important information related to LBP is CSE-MRI for assessment of paraspinal muscle quality, which may not be well characterized on routine MRI. This approach permits spatial measurements of fat fraction within the paraspinal muscles (e.g., erector spinae or multifidus muscles) [82–87]. Paraspinal muscle fat fractions showed significant correlations to isometric muscle strength as well as vertebral BM fat fractions, hence potentially reflecting the actual (patho)physiological muscle status [82, 87]. Furthermore, in a study investigating the interplay between vertebral endplate pathology and paraspinal muscle quality in patients with chronic LBP, cartilage endplate defects at the lower lumbar spine were predictive of pain when adjacent to paraspinal musculature with increased fat fractions [73]. Yet the distinct role of paraspinal musculature and particularly the fat fraction as a quantitative parameter remain largely unclear to date in patients with LBP.

While the efforts of the BACPAC Spine Imaging Working Group focus on the spine with distinct SOPs for spinal MRI protocols, it has to be acknowledged that particularly in patients suffering from chronic LBP, perceived pain may not only relate to the level of the spine. Thus, novel conceptualizations and mechanistic descriptors that involve interactions and structures beyond the lumbar spine might play an important role as well. Specifically, besides nociceptive pain (arising from ongoing input from tissue injury) and neuropathic pain (arising from injury to the peripheral or central nervous system), the concept of nociplastic pain has been described for chronic pain states that are not related to obvious nociceptor activation or neuropathy, whereas clinical and psychophysical findings suggest alterations in nociceptive function [88, 89]. As such, nociplastic pain can stem from altered pain-related sensory pathways that relate to enhanced sensitivity, and thus nociplastic pain may exist either as a comorbid or isolated state in patients with chronic pain that has been predominantly characterized as nociceptive or neuropathic [16, 88]. Particularly patients suffering from non-specific chronic LBP may show a continuum of nociceptive, neuropathic, and nociplastic components, with related increased activation of brain regions that contribute to the processing of pain and other sensory functions [88, 90–92]. Concerning patients with such mixed pain, lumbar MRI may contribute to the detection of the site that initiated pain, but imaging of central mechanisms (e.g., by functional MRI of the brain) might be needed to provide a more complete picture of the complex individual pain states.

Another relevant aspect beyond the lumbar spine might be the analysis of whole-body composition in relation to sarcopenia, which is defined as a progressive and generalized skeletal muscle disorder that involves the accelerated loss of muscle mass and function [93, 94]. While paraspinal muscle structure is routinely captured by standard lumbar MRI exams at least for some spinal levels, it is often neglected during reporting among patients with LBP, and investigations of dedicated muscle composition beyond the musculature adjacent to the spinal column are uncommon for LBP. However, it has been suggested that sarcopenia is correlated with back muscle strength and in turn also with the presence of degenerative spinal disorders [95–97]. With improved MRI hardware and software technology, it becomes possible to acquire high-quality images of large parts of the whole body in reasonable amounts of time, thus potentially making assessments of body and muscle composition beyond the direct paraspinal compartments also attractive for research in the field of chronic LBP.

## Conclusion

Management of patients suffering from LBP is challenging and requires development of diagnostic techniques to identify specific patient subgroups and phenotypes in order to customize treatment and predict clinical outcome. While lumbar MRI is frequently performed in patients with LBP, it has not yet developed into a reliable and standardized tool for these purposes. The BACPAC Spine Imaging Working Group has developed SOPs that will facilitate identification of biomarkers in lumbar MRI, which will help to support patient selection for certain treatment regimens and to predict responses to treatment. Moreover, the approach and methods outlined in this article may also serve as a recommendation for other centers performing MRI acquisitions in patients with LBP, thus potentially supporting standardization of clinical routine MRI acquisitions and reporting. Ultimately, the SOPs presented may facilitate the integration of better harmonized MRI data of the lumbar spine across studies and sites within and outside of BACPAC studies.

## Funding

This research was supported by the National Institutes of Health through the Helping to End Addiction Long-term^SM ^Initiative, or NIH Heal Initiative, as well as by the German Academic Exchange Service (Deutscher Akademischer Austauschdienst, DAAD: N.S.), the Joachim Herz Foundation (N.S.), and the Rolf W. Günther Foundation (N.S.). L.N. is principal investigator of service agreement with United Healthcare.

*Conflicts of interest:* There are no conflicts of interest to report.

## Supplement sponsorship

This article appears as part of the supplement entitled “Back Pain Consortium (BACPAC) Research Program” supported by the National Institutes of Health through the NIH HEAL Initiative under award number AR076730-01.

## Disclaimer

The content is solely the responsibility of the authors and does not necessarily represent the official views of the National Institutes of Health or its NIH HEAL Initiative.

## Abbreviations

3D: Three-dimensional
BACPAC: Back Pain Consortium
BM: Bone marrow
CSE-MRI: Chemical shift encoding-based water-fat MRI
CSF: Cerebrospinal fluid
FJ: Facet joint
FOV: Field of view
FS: Fat suppression
FSE: Fast spin-echo
LBP: Low back pain
LSTV: Lumbosacral transitional vertebrae
MRI: Magnetic resonance imaging
MRN: Magnetic resonance neurography
MRS: Magnetic resonance spectroscopy
NEX: Number of excitations
SIJ: Sacroiliac joint
SOP: Standard operating procedure
TE: Echo time
TR: Repetition time
UTE: Ultra-short echo-time
